# Optimization of LiNiCoMnO_2_ Cathode Material Synthesis Using Polyvinyl Alcohol Solution Method for Improved Lithium-Ion Batteries

**DOI:** 10.3390/nano14131096

**Published:** 2024-06-26

**Authors:** Ha Eun Kang, Tae Min Park, Sung Geun Song, Young Soo Yoon, Sang Jin Lee

**Affiliations:** 1 Department of Materials Science & Engineering, Gachon University, Seongnam-si 13120, Republic of Korea; amrorey@gachon.ac.kr; 2 Department of Advanced Materials Science & Engineering, Mokpo National University, Muan-gun 58554, Republic of Korea; 3 IL SCIENCE Co., Ltd., IL Square, 5 Saemal-ro 5-gil, Songpa-gu, Seoul-si 05808, Republic of Korea

**Keywords:** battery, cathode, lithium-ion battery, Ni-rich, synthesis

## Abstract

The growing need for lithium-ion batteries, fueled by the widespread use of electric vehicles (EVs) and portable electronic devices, requires high energy density and safety. The cathode material Li_1-x_(Ni_y_Co_z_Mn_1-y-z_)O_2_ (NCM) shows promise, but attaining high efficiency necessitates optimization of both composition and manufacturing methods. Polycrystalline LiNiCoMnO_2_ powders were synthesized and assessed in this investigation using a polyvinyl alcohol (PVA) solution method. The study examined different synthesis conditions, such as the PVA to metal ions ratio and the molecular weight of PVA, to assess their influence on powder characteristics. Electrochemical analysis indicated that cathode materials synthesized with a relatively high quantity of PVA with a molecular weight of 98,000 exhibited the highest discharge capacity of 170.34 mAh/g and a high lithium-ion diffusion coefficient of 1.19 × 10^−9^ cm^2^/s. Moreover, decreasing the PVA content, irrespective of its molecular weight, led to the production of powders with reduced surface areas and increased pore sizes. The adjustments of PVA during synthesis resulted in pre-sintering observed during the synthesis process, which had an impact on the long-term stability of batteries. The electrodes produced from the synthesized powders had a positive impact on the insertion and extraction of Li+ ions, thereby improving the electrochemical performance of the batteries. This study reveals that cathode materials synthesized with a high quantity of PVA with a molecular weight of 98,000 exhibited the highest discharge capacity of 170.34 mAh/g and a high lithium-ion diffusion coefficient of 1.19 × 10^−9^ cm^2^/s. The findings underscore the significance of optimizing methods for synthesizing PVA-based materials to enhance the electrochemical properties of NCM cathode materials, contributing to the advancement of lithium-ion battery technology. The findings underscore the significance of optimizing methods for synthesizing PVA-based materials and their influence on the electrochemical properties of NCM cathode materials. This contributes to the continuous progress in lithium-ion battery technology.

## 1. Introduction

The widespread use of products such as electric vehicles and smartphones has induced research on the corresponding energy supply sources [1,2,3]. There is active development taking place in various energy materials utilizing electrochemical reactions, particularly in the areas of environmental energy storage, electrochemical batteries, and hydrogen production and conversion systems [4,5,6]. Lithium-ion batteries are at the core of this research. LiCoO_2_ (LCO), a cathode-active material with a layered structure that determines the performance of lithium-ion batteries, is currently being commercialized because of its easy synthesis and excellent electrochemical performance [7]. However, this material is relatively expensive. Co, the most used material in lithium-ion batteries, is expensive because of its specificity and limitations. Additionally, only approximately half of the theoretical capacity (274 mAh/g) can be used with the current technology. Therefore, LCO is not suitable as a cathode-active material for medium- and large-sized lithium secondary batteries, which require both high energy densities and low prices [8,9]. Consequently, research on new cathode-active materials with high energy density and low prices that can replace LIBs is being conducted. Among the transition metals in the layered structure, Ni-rich NCM-based cathode-active materials with lower Co and higher Ni contents have received considerable attention because they can overcome the disadvantages of LCO [10,11].

Ni-rich NCM-based cathode-active materials, which are composed of Ni, Co, and Mn, have the following advantages: LiNiO_2_ (LNO) has a high capacity, LCO has excellent electrochemical performance, and LiMnO_2_ (LMO) has high stability [12,13,14,15]. However, as the Ni content increases, a phenomenon called cation mixing occurs during high-temperature synthesis [16]. This happens because Ni^2+^ (0.69 Å) has a similar size as Li^+^ (0.76 Å), causing it to occupy the layered lithium layer and form a rock salt structure of Ni-O. This hinders the movement of lithium ions, leading to adverse effects on the electrochemical performance, such as the coulombic efficiency and discharge capacity [17,18]. Therefore, to mitigate this phenomenon, various methods, such as adjusting the synthesis conditions for each NCM composition, are actively being studied [19,20,21,22].

The pore size of the cathode-active material affects the diffusion rate of Li^+^ and the electron transfer rate. This is because the smaller the pore size, the more limited the diffusion of Li ions, thereby limiting the voltage and output of the battery [18,23]. Furthermore, a higher charge/discharge capacity can be achieved as the specific surface area increases; however, this can increase side reactions between the electrode and electrolyte, resulting in a decrease in capacity and long-term performance [24,25]. To fully understand the impact of pore size on lithium-ion diffusion, various factors need to be considered. The pore size directly affects the surface area and the number of active sites available for lithium-ion interaction. Smaller pores increase the surface area, providing more active sites and shorter diffusion paths for lithium ions, thereby enhancing the diffusion rate. Additionally, the porosity of the material enhances electrolyte penetration, thereby increasing ionic conductivity. However, it is crucial to balance the pore size to maintain structural stability and prevent excessive particle aggregation, which could hinder ion transport. Therefore, optimizing the pore size is essential for maximizing lithium-ion diffusion and overall battery performance.

In the synthesis of such multi-composition ceramic materials, powder is synthesized by a solid-state reaction between powder particles. In the case of the liquid-phase method [26,27,28], a polycrystalline powder can be synthesized with a more homogeneous composition, and research on this is in progress [29]. The aggregation between each cation and the pore and particle sizes should be controlled using the liquid-phase method, which can be performed by applying the polyvinyl alcohol (PVA) polymer solution method. PVA is a polymer produced by the polymerization of vinyl alcohol and is produced by an indirect method, such as the modification of vinyl acetate, which has a similar structure owing to the instability of the vinyl alcohol monomer [20].

The deformation of PVA due to heat is small, and its aqueous solution exhibits strong adhesion to various substances, surface-active properties, and excellent emulsification and dispersing power owing to its strong protective colloidal action by adsorption. Therefore, evenly dispersing metal ions in the solution can effectively control the resulting porosity of the powder. This effect is strongly influenced by the amount of PVA added and the molecular weight, which determines the length of the polymer [26,30].

When synthesizing the cathode-active material powder using PVA, the formation of a minor phase of Li_2_CO_3_ due to residual carbon is known to degrade the battery performance [31]. Therefore, the powders must be synthesized using an appropriate amount of PVA in proportion to the total metal–molar ratio. When synthesizing polycrystalline powders using PVA, the size of the powder particles can be controlled by adjusting the amount of PVA. Batteries made with larger powder particles have been found to exhibit better electrical characteristics than those made with smaller particles [32].

In this study, the synthesis of the NCM-based cathode-active material NCM622 was attempted using the PVA solution method to obtain a cathode-active material with a homogeneous composition by forming a complex with metal ions in the polymer chain. During the synthesis process, a cathode-active material precursor was obtained, and NCM polycrystalline powder was obtained through calcination and heat treatment of the precursor. The effects of PVA addition and molecular weight control on the powder characteristics of the synthesized NCM cathode-active materials and the performance of the ion batteries were investigated.

## 2. Materials and Methods

The reagents used for the synthesis were of reagent grade and were purchased from Sigma Aldrich, USA. The precursors used for each metal component were lithium nitrate (LiNO_3_, 95%), nickel nitrate hexahydrate (Ni(NO_3_)_2_·6H_2_O, 97%), cobalt nitrate hexahydrate (Co(NO_3_)_2_ 6H_2_O, 98%), and manganese nitrate tetrahydrate (Mn(NO_3_)_2_·4H_2_O, 97%). For the PVA (polyvinyl alcohol, 99%) used, three types of PVAs with molecular weights of 9000–10,000, 31,000–50,000, and 89,000–98,000 were used to determine the effect of molecular weight. The respective metal nitrates (Li, Ni, Co, and Mn) were completely dissolved in deionized water using a magnetic stirrer with a Li:Ni:Co:Mn ratio of 1.05:0.6:0.2:0.2. (Table 1). PVA was added at a ratio of 4:1 or 16:1 with respect to the average atomic weight of the four metal cations, as listed in Table 2. The average valency of Ni, Co, and Mn is +2. Because PVA has two more electrons per –(OH) group, the actual ratio when PVA is added at a 4:1 molar ratio is estimated to be 8:1 because of the difference in charge. Therefore, to achieve a 4:1 ratio, PVA was added at a PVA:M^2+^ ratio of 2:1, considering that the molecular weight of the PVA monomer is 44 [27]. The atomic ratio becomes an important factor when adding PVA because the –(OH) groups of PVA in solution can affect the dispersion of metal cations [33,34].

A sol-type solution was prepared by mixing a solution containing metal nitrates with a 5 wt% PVA solution, which was stirred at 150 rpm using a magnetic stirrer at 200 °C. As the moisture evaporated, the mixture transformed into a high-viscosity slurry, and the metal nitrates decomposed, leading to the generation of NO_x_ gas and causing the solution to swell. The resulting gel-like precursor was stirred thoroughly until no more gas was generated and then dried in an oven at 200 °C for 12 h. Subsequently, the precursor was subjected to a 1-h pyrolysis process at 500 °C, during which the polymeric chains of PVA were thermally decomposed, yielding a black powder precursor. This precursor was then heat-treated at 850 °C for 20 h to synthesize polycrystalline LiNi_0_._6_Co_0_._2_Mn_0_._2_O_2_ powder.

A solution containing metal nitrates and a 5 wt% PVA solution were mixed to form a sol-type solution, which was stirred using a magnetic bar at 150 rpm in a mixer at 200 °C. As the water evaporated, a high-viscosity slurry was formed, and the metal nitrates decomposed, resulting in the generation of NO_x_ gas and swelling of the mixture. The gel-like precursor was stirred sufficiently until no more gas was generated and then dried at 200 °C in an oven for 12 h. Subsequently, the black powder precursor was obtained as the polymer chains of PVA decomposed upon heating at 500 °C for 1 h. The powder precursor was then subjected to heat treatment at 850 °C for 20 h to synthesize the polycrystalline LiNi_0_._6_Co_0_._2_Mn_0_._2_O_2_ powder. The crystal structure of the synthesized powder was analyzed using X-ray diffraction (XRD, X’pert–pro MPD, PAN alytical, Almelo, The Netherlands) at a scan speed of 4°/min, 40 kV, and 30 mA. Scanning electron microscopy (SEM; Regulus8230, Hitachi, Tokyo, Japan) was used for microstructural and compositional analyses of the synthesized powder. The thermal analysis of the precursor powder was carried out using a thermal analyzer (SDT650, Ta Instruments, New Castle, DE, USA) at a heating rate of 10 °C/min in air up to 1000 °C. The specific surface area of the synthesized powder was measured using an automated surface and pore size analyzer (Quadrasorb SI-Kr; Quantachrome Instruments, Boynton Beach, FL, USA). To ensure the reliability and accuracy of specific surface area and pore diameter measurements, two samples prepared using the same method were measured twice, and the average values were obtained. The electrochemical properties were evaluated using a half-cell prepared with a 2032-coin cell. Half-cells were prepared in a glove box under an Ar (99.999%) atmosphere with controlled levels of oxygen and moisture below 1 ppm. The electrodes were prepared by mixing the active material, conductive agent (Super-P), and binder (PVdF, polyvinylidene difluoride) in a ratio of 8:1:1 (by weight) using the solvent NMP (N) and coating the slurry onto an aluminum foil. The electrode loading and density were controlled, and the electrode was dried at 70 °C for 3 h before cell assembly. Lithium metal was used as the anode, a polypropylene (PP) membrane was used as the separator, and 1 M LiPF_6_ in EC/DMC (1:1) was used as the electrolyte. The charge/discharge curves were assessed by testing the half-cell within a voltage range of 2.5–4.3 V and at 0.5 C-rate at room temperature using a battery cycler (WBCS–3000). The battery cycling tests were conducted at a constant temperature of 25 °C to maintain the consistency and reliability of the electrochemical performance data. Electrochemical impedance spectroscopy (EIS) analysis was performed on a Biologic VMP3 system using an AC amplitude of 10 mV in the range from 100 kHz to 0.01 Hz at room temperature.

## 3. Results

**Figure 1:**
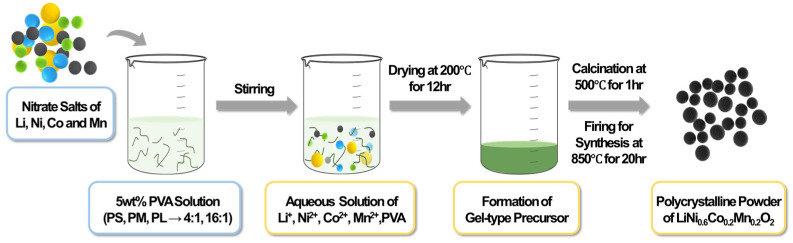
Schematic of synthesis of the LiNi_0_._6_Co_0_._2_Mn_0_._2_O_2_ powder using the PVA solution method.

The synthesis of the cathode-active material involved the use of citric acid with a carboxyl (–COOH) group in the PVA solution. This synthesis method is similar to the Pechini method for obtaining a precursor or the polymer method for obtaining a precursor using poly(acrylic acid) with a carboxyl group. Alternatively, the precursor can be obtained using polyvinyl alcohol, which is a polymer with a hydroxyl group (–OH) instead of a carboxyl group [35]. Because the PVA content was high during the synthesis process, volume expansion occurred during the stirring process, probably caused by the generation of NOx gas. The higher the PVA content, the higher the viscosity of the mixed solution, which occurred continuously until the NOx gas generated from the metal salt in the solution had completely dried. In this case, a soft, porous precursor was produced [35]. The procedure for synthesizing the LiNi_0_._6_Co_0_._2_Mn_0_._2_O_2_ powder using the PVA solution method is shown in Figure 1. Without using PVA, obtaining a precursor with a uniform composition is difficult because the constituent metal components precipitate separately, owing to the differences in solubility when water is removed from the aqueous solution. However, by the time moisture is removed from the PVA-dissolved solution, the metal ions in the solution have already formed complexes with the hydroxyl groups of the polymer chains; therefore, even after water removal, relatively uniform dispersion of cations can be maintained.

In this study, the aim is to clarify how the drying process, facilitated by the solvent and PVA binder, influences the formation of porosity and subsequently alters the electrochemical characteristics. During the initial stages of the drying process, NCM particles become saturated with a mixture of solvent and dissolved PVA binder, filling all the pores. As the drying process progresses, the NCM particles come closer together, and this mixture continues to shrink until it is fully dried. The solvent–PVA mixture within the large interparticle pores between particles gradually empties through capillary transport, resulting in smaller pores that transport moisture toward the electrode surface. In contrast, the smaller intraparticle pores within the NCM particles remain filled due to significantly higher capillary pressures. The drying process is considered complete when all solvent and polyvinyl alcohol (PVA) binders have been removed from within the NCM particles. As a result, both large and small pores are formed within the NCM cathode material, allowing access to a greater number of chemical reaction sites.

**Figure 2:**
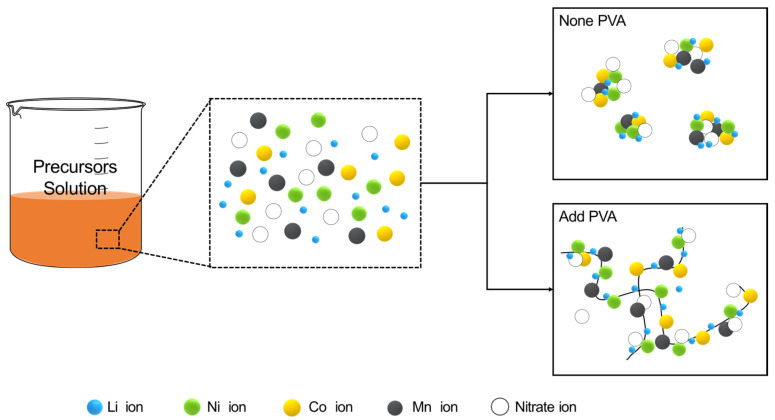
Schematic for dispersion mechanism of metal cations by PVA solution method.

The dispersion state of the metal cations within the dry gel with an appropriate amount of PVA is shown in Figure 2. The long-chain PVA molecules present in the aqueous solution evenly dispersed the metal ions to yield a gel-state precursor without aggregation. When heated at 500 °C, the PVA polymer undergoes thermal decomposition, resulting in a porous powder precursor. This powder was then subjected to prolonged heat treatment to synthesize an active cathode material. In the PVA solution method, the degree of dispersion can vary depending on the molecular weight and amount of PVA added, enabling the control of physical properties such as particle size, porosity, and specific surface area of the synthesized powder. In comparison to the Pechini method, the key distinction lies in how cations are dispersed in the solution. In the Pechini method, cations are dispersed through the polymerization of citric acid. On the other hand, in the case of PVA, cations are adsorbed and dispersed by the –(OH) group located at the end of the monomer of the dissolved PVA polymer. Consequently, the dispersion of cations in the PVA method is more influenced by the amount and molecular weight of PVA used in the process.

**Figure 3:**
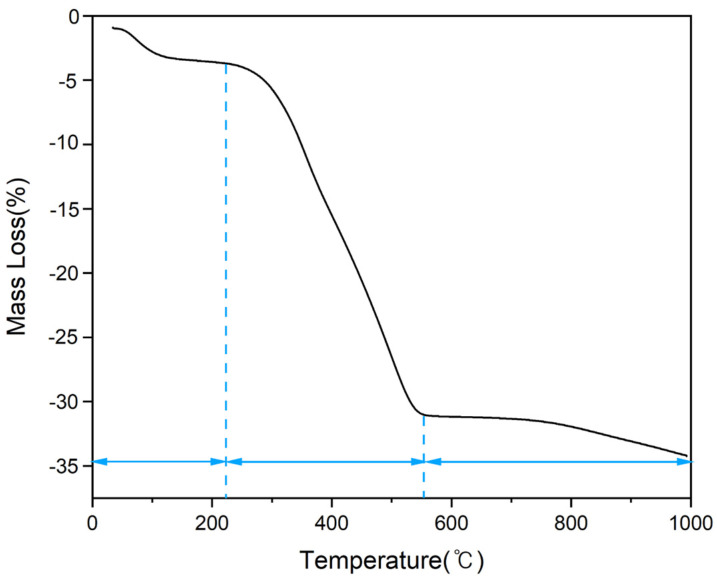
TGA curve of NCM precursor.

Thermogravimetric analysis (TGA) results for the NCM precursor gel synthesized using PVA with a molecular weight of 50,000 are shown in Figure 3. The Initial Decomposition Temperature (IDT) and Final Residue (FR) were determined from the TGA curve to assess the thermal stability and composition of the precursor materials. The IDT indicates the temperature at which the material begins to decompose, while the FR represents the remaining mass after completing thermal decomposition, providing insights into the purity and stability of the synthesized powders. The precursor gel exhibited a weight loss of about 5% due to the thermal decomposition of residual NO_x_ gas up to 200 °C, and a rapid weight loss of around 25% occurred between 200 and 550 °C due to the thermal decomposition and oxidation of PVA, resulting in the removal of carbon.

In our study, the synthesized NCM samples were categorized based on their molecular weight (M.W.), with distinctions made among small, medium, and large molecular weights. Additionally, various mixing ratios were employed in the preparation, specifically denoted as 4:1 and 16:1. The information for each sample, according to the molecular weight and PVA content, is presented in Table 3.

PVA possesses diverse chemical and physicochemical properties, making it a versatile additive that can be incorporated into cathode materials through various synthesis methods. Its high solubility and flexibility are particularly advantageous for controlling the particle size and structure of cathode materials.

**Figure 4:**
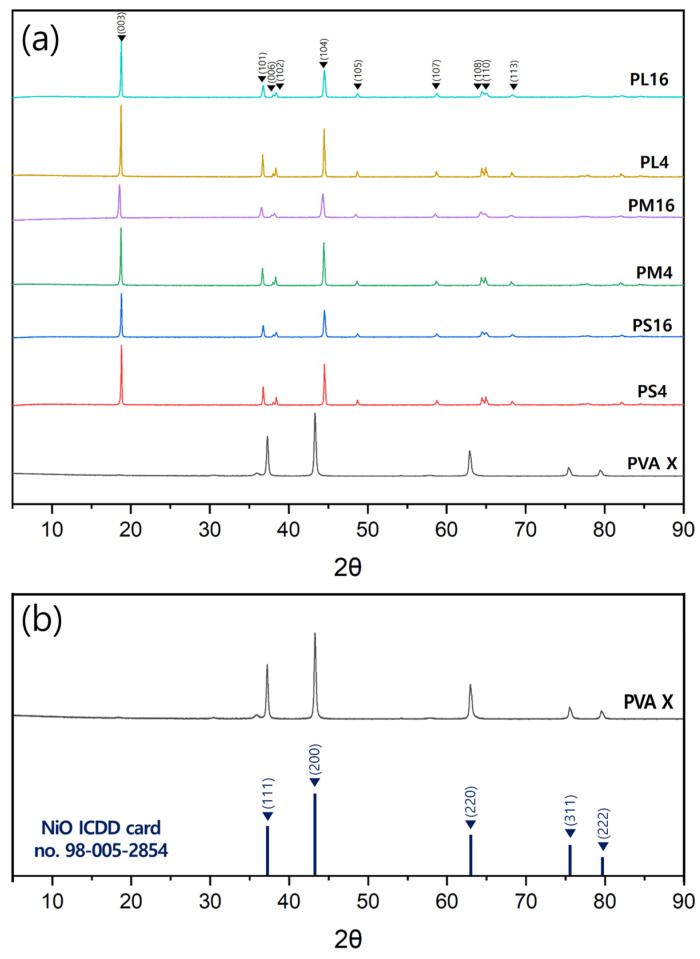
X-ray diffraction patterns of (**a**) LiNi_0_._6_Co_0_._2_Mn_0_._2_O_2_ powders synthesized at different temperatures and (**b**) PVA X samples.

The X-ray diffraction pattern of the LiNi_0_._6_Co_0_._2_Mn_0_._2_O_2_ powder heat-treated at 850 °C is shown in Figure 4a. Except for the powder synthesized without PVA, all synthesized powders showed only the peak of LiNi_0_._6_Co_0_._2_Mn_0_._2_O_2_, and no other minor peaks were observed. The generation of the crystal phase was not affected by the type or amount of PVA. In samples with added PVA, the analysis revealed that the peak with the highest intensity was observed at approximately 19°, and two lines were located at 44°, indicating a crystal structure of the $R\bar{3}$m space group [25]. The layered structure is well-defined, as the (006) and (102) peaks and the (108) and (110) peaks are clearly separated from each other [22,36,37,38].

The XRD data for the powders synthesized without the addition of PVA are also presented to investigate the influence of PVA. Figure 4b presents the X-ray diffraction (XRD) analysis of the PVA × sample, indicating the presence of NiO particles (according to ICDD card number 98-005-2854). This observation suggests that without the addition of PVA, the complete synthesis of LiNi_0.6_Co_0.2_Mn_0.2_O_2_ does not occur, leading to the presence of NiO as an intermediate phase. Furthermore, the absence of layered 622 NCM formation in the PVA × sample is attributed to the heterogeneous nature of the synthesis reaction between the three materials. This heterogeneity causes separation and severe aggregation during the drying of the solution precursor due to differences in cation density, further hindering the synthesis process.

To investigate the extent of cation mixing, a phenomenon where Li^+^ (0.76 Å) and Ni^2+^ (0.69 Å), which have similar ionic radii, exchange their positions in the crystal lattice, the I(003)/I(004) ratio was calculated and is shown in Table 4. Typically, when the I(003)/I(004) value exceeds 1.2, the layered structure of the cathode material is considered to be well-formed. Therefore, the PS, PM, and PL samples were well synthesized in a layered structure. In all groups, the I(003)/I(004) values increased as the amount of added PVA decreased. Notably, for the high-molecular-weight PVA used in the PL group, the PL16 sample exhibited a significant difference in the I(003)/I(004) value of 1.87, depending on the PVA content. These results suggest that PVA with a higher molecular weight is more advantageous for layer formation when a relatively small amount is added [39].

XRD analysis was performed to determine the effects of the molecular weight and the amount of PVA added to the primary particle size of the synthesized LiNi_0.6_Co_0.2_Mn_0.2_O_2_ powder. Based on the XRD data, the primary particle sizes of the powders were calculated using the Scherrer Equation [35,40] and are shown in Table 5. The size of the crystallites can also affect the electrochemical properties of NCM cathode materials. A smaller crystallite size is expected to lead to a higher surface area in relation to its volume, potentially enhancing electrochemical reactions that occur at the material’s surface.

**Figure 5:**
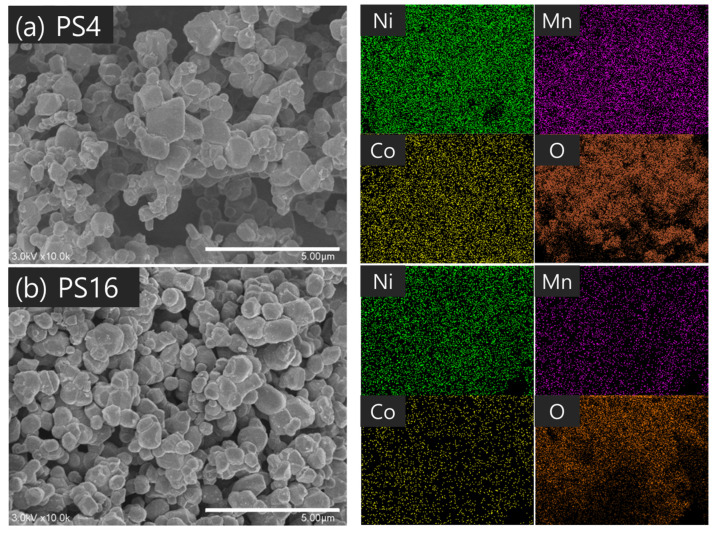
SEM images and EDS analysis of LiNi_0_._6_Co_0_._2_Mn_0_._2_O_2_ powders synthesized under the conditions of (**a**) PS4 and (**b**) PS16.

**Figure 6:**
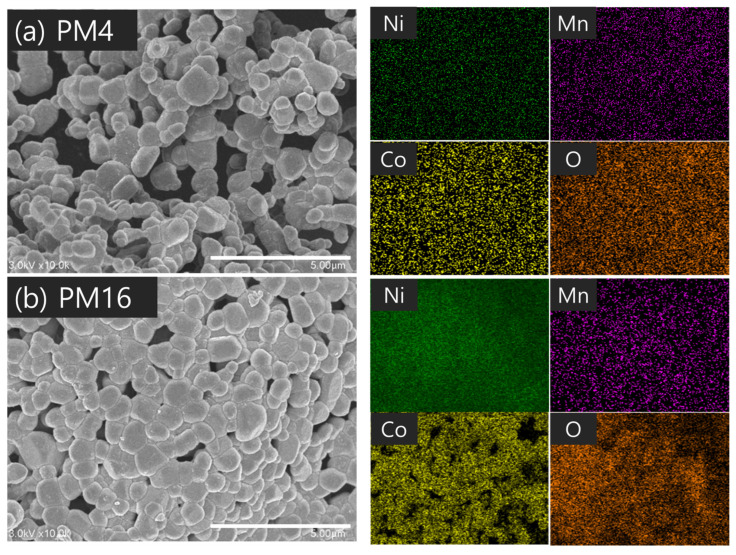
SEM images and EDS analysis of LiNi_0_._6_Co_0_._2_Mn_0_._2_O_2_ powders synthesized under the conditions of (**a**) PM4 and (**b**) PM16.

**Figure 7:**
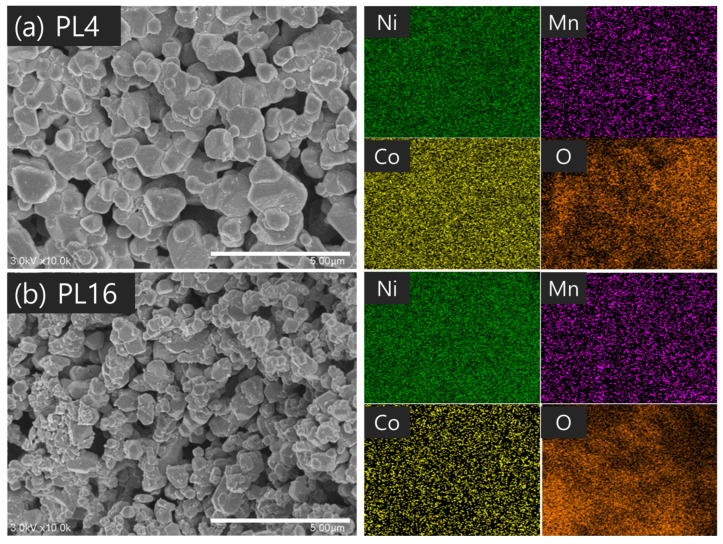
SEM images and EDS analysis of LiNi_0_._6_Co_0_._2_Mn_0_._2_O_2_ powders synthesized under the conditions of (**a**) PL4 and (**b**) PL16.

When PVA with a molecular weight of 10,000 was used, the primary particle size was relatively large, as observed for the PS sample group. In contrast, the PM and PL groups exhibited similar particle sizes of 60–61 nm for the primary particles. Proper control of the molecular weight and content of PVA can minimize the aggregation of cathode material particles and reduce the size of the crystals produced during synthesis. When a small amount of PVA with a low molecular weight was added (PS16), the effect of the PVA addition was negligible; as a result, the primary particle size was observed to be large. The microstructures of the synthetic powders according to the molecular weight were confirmed, as shown in Figure 5, Figure 6 and Figure 7. Particle size was larger for a PVA mixing ratio of 4:1 than for 16:1. Considering that the primary particle size was approximately 60 nm, the secondary particle size of the powder observed by SEM was due to the aggregation of many primary particles. As shown in Figure 5, the powder particles (secondary particles) were relatively small in PS16, which had a low PVA molecular weight and mixing ratio, indicating that the amount of aggregation of the primary particles was small. However, local particle aggregation was also observed. As the molecular weight and mixing ratio increase, powder aggregation is not expected to increase significantly, but the aggregation of primary particles is affected to some extent. In Figure 7, slight pre-sintering occurred in PL16, which had a PVA molecular weight of 98,000. This is believed to be due to the inhibition of local particle aggregation, the occurrence of a relatively homogeneous distribution, and the large contact area between the particles owing to their growth.

**Figure 8:**
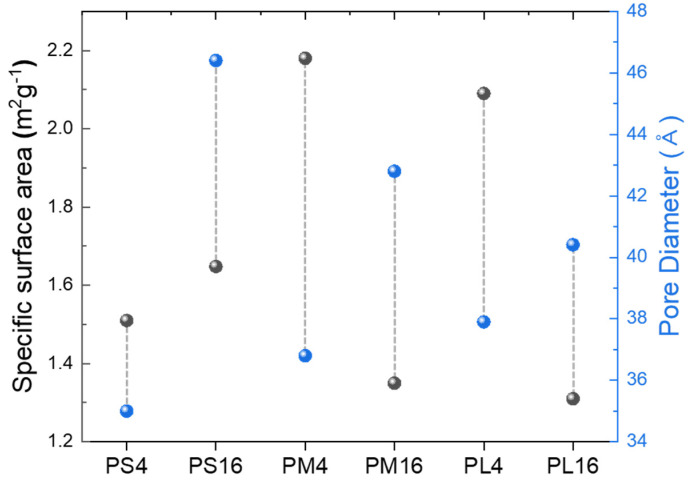
BET and pore diameter according to PVA M.W. and mixing ratio.

The Brunauer–Emmett–Teller (BET) method was used to determine the effects of the specific surface area and pore size of the powder on the molecular weight and content of PVA, as shown in Figure 8 [41]. When the PVA molecular weight was 10,000, both BET value and pore size increased with a decrease in PVA content, as seen in Figure 5, which is attributed to the reduction in particle size with a low amount of PVA. In contrast, for PVA molecular weights of 50,000 and 98,000, a decrease in the PVA content led to an increase in the pore size and a decrease in the BET value. Generally, the presence of small pores in a powder leads to an increase in the specific surface area.

The results suggest that the particle size of the powder is mainly influenced by the aggregation of primary particles, and as the molecular weight and mixing amount of PVA increase, the aggregation of primary particles is affected. Pre-sintering occurs due to well-formed particle agglomeration and increased contact between particles. However, this trend was not observed for the PS sample, which is thought to be due to the relatively low formation of bubbles during the drying process of the precursor solution owing to the low molecular weight of PVA. The BET values of the PM4 and PL4 samples with a high PVA content of 4:1 were observed to be high, indicating a relatively higher inclusion of pores in the powder. However, the PL16 sample exhibited a relatively lower BET surface area than PL4, with a pore size of 40.41 Å. This is attributed to the lower PVA content and the presence of larger pores in the powder.

**Figure 9:**
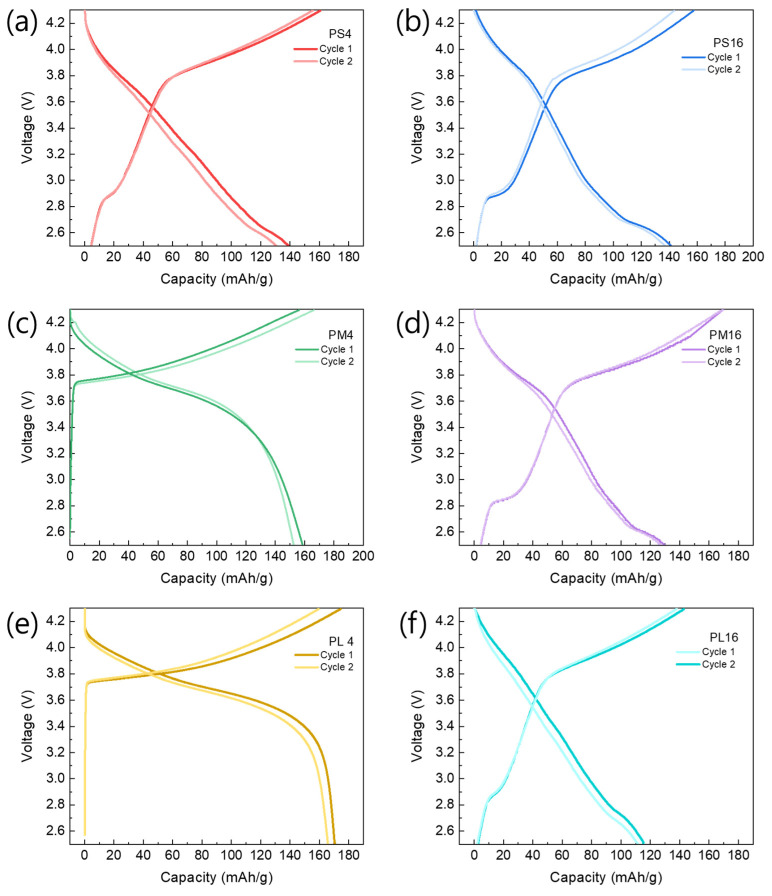
Charge/discharge curves of (**a**,**b**) PS, (**c**,**d**) PM, and (**e**,**f**) PL cathode samples in the 2.5–4.3 V vs. Li^+^/Li range at 0.5 C.

The cycle characteristics of the LiNi_0_._6_Co_0_._2_Mn_0_._2_O_2_ powder according to the changes in PVA in the range of 2.5–4.3 V vs. Li^+^/Li are shown in Figure 9. This section examines the impact of the molecular weight and PVA content on the electrochemical performance of the synthesized NCM cathode material. We conducted a comparison and analysis of the cycle characteristics of NCM samples with varying PVA parameters. This section examines the impact of the molecular weight and PVA content on the electrochemical performance of the synthesized NCM cathode material. We compare and analyze the cycle characteristics of NCM samples with varying PVA parameters.

Figure 9 depicts the cycle characteristics of the NCM samples that were synthesized using PVA with different molecular weights: PS (low), PM (medium), and PL (high). The initial capacity of the PS4 (138.63 mAh/g) is marginally lower compared to that of the PM4 (158.67 mAh/g) and PL4 (170.34 mAh/g). During cycling, the PS4 demonstrates a more significant decrease in capacity compared to the PM4 and PL4. In contrast, samples with a 16:1 PVA mixing ratio (PS16, PM16, and PL16) show that PS16 possesses the highest initial capacity, reaching 141.12 mAh/g, surpassing both PM16 (124.63 mAh/g) and PL16 (115.82 mAh/g). Furthermore, PS16 demonstrates consistent capacity retention during cycling, while PM16 and PL16 exhibit more pronounced capacity degradation.

**Figure 10:**
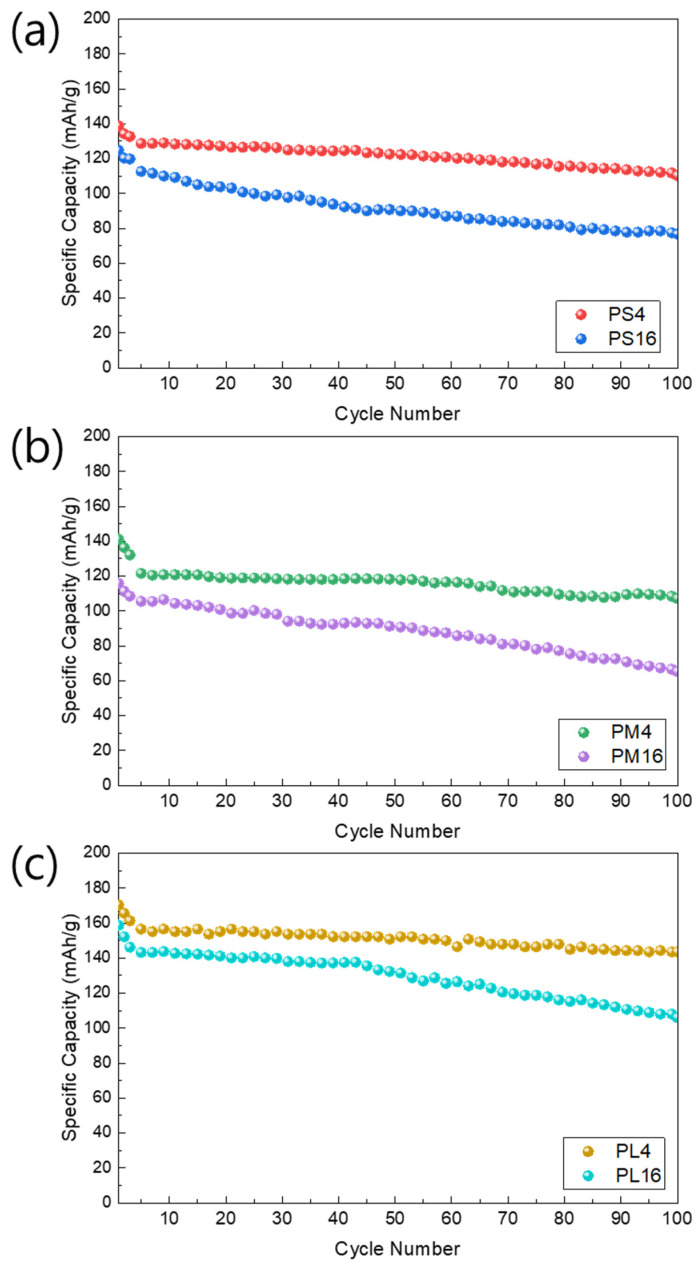
Plot of specific capacity (*y*-axis) versus cycle number of (**a**) PS, (**b**) PM, and (**c**) PL samples.

These results indicate that higher PVA content leads to superior performance when the molecular weight of PVA remains constant. However, it is essential to note that maintaining a consistent ratio between the active material and PVA can result in varying characteristics. Further investigation examines the impact of PVA content by comparing samples with different PVA mixing ratios (4:1 vs. 16:1) while maintaining a medium molecular weight (PM). In Figure 10, PM4 (4:1) shows a higher initial capacity (158.67 mAh/g) compared to PM16 (16:1), which has an initial capacity of 124.63 mAh/g. Additionally, PM4 exhibits more favorable capacity retention during cycling than PM16. These findings suggest that, in the context of medium molecular weight PVA, a lower PVA content (4:1) contributes to improved initial capacity and cycle stability compared to a higher PVA content (16:1).

The electrochemical performance of NCM cathode materials is primarily influenced by pore structure and specific surface area. The specific surface area and pore size of NCM powders can be analyzed using the BET method, which may affect these electrochemical characteristics. The presence of small pores increases the specific surface area, providing more reaction sites at the electrode–electrolyte interface and thereby enhancing the initial capacity. For instance, the PL4 sample exhibited high initial capacity and consistent capacity retention, which could be attributed to the optimized pore structure resulting from a high PVA content. Conversely, large pores can negatively impact long-term cycling performance. The PS16 sample, with its larger pore size, showed a lower capacity retention rate compared to the PS4 sample, which had smaller pore sizes. Large pores can reduce structural stability, leading to particle breakdown and electrode degradation during cycling. Additionally, a higher specific surface area facilitates the insertion and removal of lithium ions during cycles, positively influencing capacity retention.

Figure 10 depicts the comparison of capacity changes over 100 charge/discharge cycles for each sample, emphasizing the notable influence of variations in PVA molecular weight and content on cyclic stability. The PS4 and PS16 cells exhibit consistent capacity retention up to the second cycle, with PS4 retaining 79.49% of its original capacity even after 100 cycles. The observation suggests that the PS4 sample, which was synthesized using relatively low molecular weight PVA and high content, demonstrates robust electrochemical characteristics from the initial stages. In contrast, PM4 and PM16 demonstrate a significant decline in capacity after 100 cycles, with PM16 showing a particularly low-capacity retention rate of only 61.48%. This indicates that the cyclic stability of these samples is adversely affected by intermediate molecular weight and PVA content.

The PL4 and PL16 samples exhibit intriguing results with regard to capacity retention. PL4 demonstrates a high-capacity retention rate of 84.24% over 100 cycles, suggesting that the utilization of a relatively high PVA molecular weight and low content contributes to improved cyclic stability. Nevertheless, PL16 demonstrates a reduced capacity retention rate of 56.31%, indicating that increased PVA molecular weight and content could be associated with capacity deterioration. To elucidate these observations, it is crucial to consider the impact of polyvinyl alcohol (PVA) molecular weight and content on the pore size distribution within the NCM cathode material. The use of high-molecular-weight PVA (PL) can lead to larger pore sizes, which may facilitate the insertion and removal of Li^+^ ions, thereby potentially increasing the capacity and stability of the system [14,42,43].

In summary, our findings underscore the significant impact of both the molecular weight and content of PVA on the electrochemical properties of NCM cathode materials. The utilization of high-molecular-weight PVA (PL) and a reduced PVA content (4:1) shows potential for enhancing the efficacy of NCM cathodes. Overall, pore structure and specific surface area affect the electrochemical performance of NCM cathode materials, resulting in varying cycle characteristics. Furthermore, high PVA molecular weight and content can increase particle aggregation and promote sintering, thereby enhancing structural stability, which is beneficial for long-term cycling performance.

**Figure 11:**
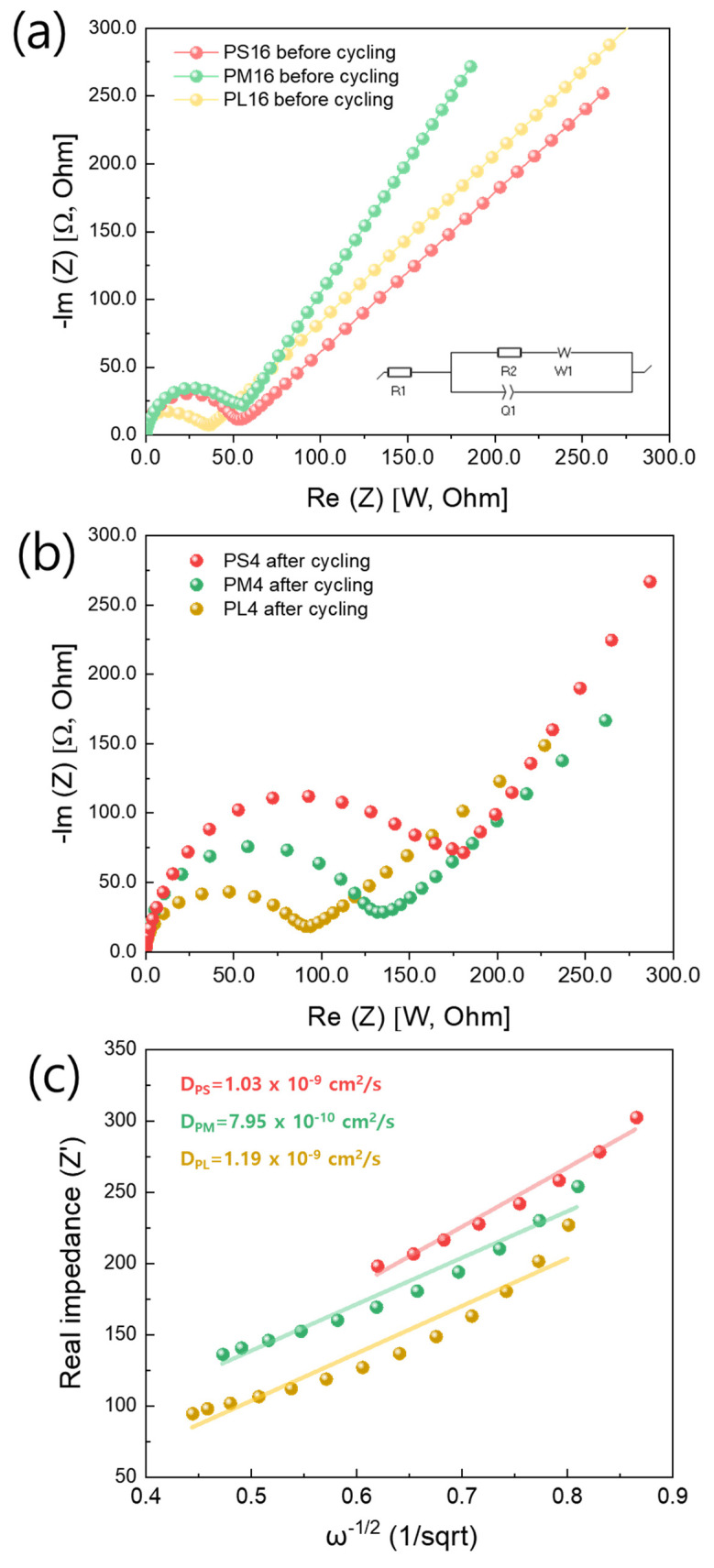
(**a**,**b**) Nyquist plots of PS16, PM16, and PL16 cathode before and after 100 cycles, (**c**) relationship between imaginary resistance (Z’) and inverse square root of angular speed (ω^−1/2^).

The electrochemical kinetics of the PS16, PM16, and PL16 samples were thoroughly examined using Electrochemical Impedance Spectroscopy (EIS) post-cycling, as illustrated in Figure 11 (before and after 100 cycles, 0.5 C). EIS provides invaluable insights into the electrochemical processes within the material, allowing for a deeper understanding of its performance characteristics. Each Nyquist plot was composed of a high-frequency semicircle and a Warburg tail region followed by a steeply sloping line in the low-frequency region. [44] The semicircle indicates the charge transfer resistance (Rct), whereas the straight line in the low-frequency region reflects the Warburg impedance (Wo), which is related to Li+ diffusion in the bulk of the electrode. Figure 11a,b shows a Nyquist plot, effectively illustrating the impedance characteristics of each sample. The impedance values follow the order of PS4 > PM4 > PL4. This observation suggests that PS4 has the highest impedance among the samples, followed by PM4 and PL4. It is worth noting that impedance is a direct measure of electrical resistance, and higher impedance can potentially lead to reduced electrical efficiency.

Following the cycling process, the impedance values demonstrate a notable elevation in comparison to the pre-cycling conditions. This increase indicates a substantial rise in charge transfer resistance (R_ct_) post-cycling, which is likely due to electrode degradation, increased interface resistance, or structural changes at the electrolyte–electrode interface induced by cycling. The observed increase in post-cycling impedance signifies a decrease in electrical conductivity and efficiency within the electrode material. Higher impedance has a deleterious effect on the performance of lithium-ion batteries, leading to a reduction in charge–discharge efficiency and the potential for overall degradation in battery performance over extended cycling periods. In addition, the Li^+^ diffusion coefficient (D_Li_^+^) for the samples was computed using the following formula: [45,46,47,48]

*R* = the gas constant (8.314 J/mol·K)

*T* = the absolute temperature (K)

*A* = the surface area of the electrode (m^2^)

*n* = the number of electrons per molecule during oxidization

*C* = the concentration of Li^+^ (mol/L)

*σ* = the Warburg factor

*F* = the Faraday constant (96,485 C/mol)

$$D_{{Li}^{+}diffusion\;}=\frac{R^{2}T^{2}}{{2A}^{2}n^{4}c^{2}\sigma^{2}F^{4}}$$

The provided equation for the Li^+^ diffusion coefficient is a representation that relates the diffusion coefficient to various physical and electrochemical parameters. The theory behind such equations often stems from electrochemical kinetics and transport phenomena within active materials. The Warburg coefficient was calculated from the plot of low frequency (Warburg) real impedance (Z_real_) vs. 1/sqrt (ω^−1/2^). ω is the angular frequency calculated from the impedance data. With the Warburg coefficient at hand, we proceeded to compute the lithium diffusion coefficient for each sample. The values obtained are as follows in Figure 11b. These values represent the rate at which lithium ions diffuse within the respective samples, offering critical insights into their electrochemical performance and stability.

Based on the results, the lithium diffusion constants for the PS4, PM4, and PL4 samples were determined to be 1.03 × 10^−9^ cm^2^/s, 7.95 × 10^−10^ cm^2^/s, and 1.19 × 10^−9^ cm^2^/s, respectively. Specifically, the PS4 demonstrates the highest real impedance, followed by the PL4 and PM4. These findings provide valuable insights into the electrochemical characteristics of the samples, with PS4 notably demonstrating the highest impedance resistance. In summary, the combined information from Figure 11a,b collectively elucidates the intricate relationship between impedance resistance, frequency, and diffusion constants for the PS4, PM4, and PL4 samples. These insights provide a comprehensive understanding of the electrochemical characteristics of the materials under study, with PS4 demonstrating the highest impedance resistance.

Nanostructured NCM particles have demonstrated notable benefits in energy storage applications, as evidenced by prior research. These advantages include the facilitation of rapid solid-state diffusion and charge transport, surpassing those of dense active materials [49,50]. Expanding on these findings, our study sought to analyze the pore size and surface area of NCM materials by varying the PVA molecular weight and content. Subsequently, we performed electrochemical tests to evaluate their influence on the electrochemical properties and battery performance of the materials.

Consequently, we were able to effectively illustrate the impact of PVA additive conditions on the electrochemical characteristics and battery performance of the active material. In particular, the addition of high-molecular-weight PVA at a ratio of 4:1 (PL4) resulted in the optimization of pore size and electrochemically active surface area. This optimization has contributed to improved electrochemical stability, ultimately enhancing the long-term stability and performance of the battery.

## 4. Conclusions

This study centered on the production of layered LiNi_0.6_Co_0.2_Mn_0.2_O_2_ cathode materials through the PVA solution method, which entailed a meticulous procedure encompassing the elimination of polymer residues and heat treatment at 850 °C for 20 h. The focus of the study was to examine the molecular weight and content of PVA as crucial factors, which resulted in several significant discoveries. Initially, it was noted that the studied samples demonstrated the most outstanding electrochemical performance when high molecular weight PVA, particularly with a molecular weight of 98,000, was used, and the PVA content was increased. The optimal condition successfully regulated particle aggregation and size, ultimately leading to a greater initial capacity. Nevertheless, it was observed that powders possessing a high specific surface area led to an increase in side reactions during charge/discharge processes, leading to a decline in electrochemical performance. Moreover, our research emphasizes the crucial significance of optimizing the molecular weight and content of PVA to control the specific surface area and pore size within the powder. This optimization ultimately aids in the advancement of next-generation cathode materials with enhanced electrochemical characteristics. Furthermore, a comprehensive investigation was carried out on the impedance and lithium diffusion constants, encompassing the PS4, PM4, and PL4 samples. The unfavorable performance of batteries is directly associated with the increased impedance and reduced lithium-ion diffusion coefficients in cathode materials. This discovery makes a significant contribution to the optimization of NCM synthesis for advanced lithium-ion batteries. The PVA solution method utilized in our study is notably regarded as an effective and cost-efficient approach. The diverse facets of our study are anticipated to propel progress in lithium-ion battery technology. There are several constraints within the current study. Firstly, additional research is needed to evaluate the scalability of the PVA solution method for large-scale production, as unanticipated variables may arise during industrial scaling. Achieving consistent quality requires the advancement of automation technologies and process monitoring systems. Secondly, a more comprehensive investigation is necessary to understand the influence of the microstructure of PVA-formed NCM on its interfaces with liquid or solid electrolytes. This includes interfacial stability, electrochemical interactions, and thermal and mechanical stability throughout charge/discharge cycles. Enhancing compatibility with solid electrolytes requires advancements in surface modification techniques and the creation of new electrolyte materials. Future studies should prioritize these aspects to optimize performance and ensure the long-term stability of lithium-ion batteries.

## Figures and Tables

**Table 1:** Specifications of materials used.

Material	Chemical Formula	Purity	Molecular Weight/Form	Remarks
Lithium nitrate	LiNO_3_	95%	Solid	Sigma Aldrich, St. Louis, MO, USA
Nickel nitrate hexahydrate	Ni(NO_3_)_2_·6H_2_O	97%	Solid	Sigma Aldrich, St. Louis, MO, USA
Cobalt nitrate hexahydrate	Co(NO_3_)_2_·6H_2_O	98%	Solid	Sigma Aldrich, St. Louis, MO, USA
Manganese nitrate tetrahydrate	Mn(NO_3_)_2_·4H_2_O	97%	Solid	Sigma Aldrich, St. Louis, MO, USA
Polyvinyl alcohol (PVA)	(C_2_H_4_O)n	99%	Three types: 9000–10,000, 31,000–50,000, 89,000–98,000	Sigma Aldrich, St. Louis, MO, USA

**Table 2:** Relative amounts of metal nitrate and PVA polymer according to PVA mixing ratio.

Mixing Ratio	Total Metal Nitrate (g)	PVA (g)
4:1	30	3.5563
16:1	30	0.8890

**Table 3:** Sample name according to PVA M.W. and mixing ratio.

Sample	PVA M.W.	PVA Mixing Ratio
PS4	9000~10,000	4:1
PS16		16:1
PM4	31,000~50,000	4:1
PM16		16:1
PL4	89,000~98,000	4:1
PL16		16:1

**Table 4:** I(003)/I(104) data using XRD results for LiNi_0.6_Co_0.2_Mn_0.2_O_2_.

	I(003)	I(104)	I(003)/I(104)
PS4	27,364.93	19,642.41	1.393155
PS16	20,513.67	13,510.91	1.544058
PM4	26,813.77	20,702.49	1.295195
PM16	16,222.53	12,186.49	1.479811
PL4	32,518.86	22,969	1.415772
PL16	26,205.09	13,946.61	1.878958

**Table 5:** Crystallite size of primary particles of the synthesized LiNi_0_._6_Co_0_._2_Mn_0_._2_O_2_ powder according to PVA M.W and mixing ratio.

Sample	PVA M.W	PVA Mixing Ratio	Crystallite Size (nm)
PS4	9000~10,000	4:1	63.48
PS16		16:1	65.38
PM4	31,000~50,000	4:1	61.3
PM16		16:1	61.03
PL4	89,000~98,000	4:1	60.14
PL16		16:1	61.39

## Data Availability

The original contributions presented in the study are included in the article, further inquiries can be directed to the corresponding authors.
